# CNS Cell Distribution and Axon Orientation Determine Local Spinal Cord Mechanical Properties

**DOI:** 10.1016/j.bpj.2015.03.039

**Published:** 2015-05-05

**Authors:** David E. Koser, Emad Moeendarbary, Janina Hanne, Stefanie Kuerten, Kristian Franze

**Affiliations:** 1 Department of Physiology, Development and Neuroscience, University of Cambridge, Cambridge, United Kingdom; 2 Department of Anatomy I, University of Cologne, Cologne, Germany; 3 Department of Anatomy and Cell Biology, University of Wuerzburg, Wuerzburg, Germany

## Abstract

Mechanical signaling plays an important role in cell physiology and pathology. Many cell types, including neurons and glial cells, respond to the mechanical properties of their environment. Yet, for spinal cord tissue, data on tissue stiffness are sparse. To investigate the regional and direction-dependent mechanical properties of spinal cord tissue at a spatial resolution relevant to individual cells, we conducted atomic force microscopy (AFM) indentation and tensile measurements on acutely isolated mouse spinal cord tissue sectioned along the three major anatomical planes, and correlated local mechanical properties with the underlying cellular structures. Stiffness maps revealed that gray matter is significantly stiffer than white matter irrespective of directionality (transverse, coronal, and sagittal planes) and force direction (compression or tension) (K_g_= ∼130 Pa vs. K_w_= ∼70 Pa); both matters stiffened with increasing strain. When all data were pooled for each plane, gray matter behaved like an isotropic material under compression; however, subregions of the gray matter were rather heterogeneous and anisotropic. For example, in sagittal sections the dorsal horn was significantly stiffer than the ventral horn. In contrast, white matter behaved transversely isotropic, with the elastic stiffness along the craniocaudal (i.e., longitudinal) axis being lower than perpendicular to it. The stiffness distributions we found under compression strongly correlated with the orientation of axons, the areas of cell nuclei, and cellular in plane proximity. Based on these morphological parameters, we developed a phenomenological model to estimate local mechanical properties of central nervous system (CNS) tissue. Our study may thus ultimately help predicting local tissue stiffness, and hence cell behavior in response to mechanical signaling under physiological and pathological conditions, purely based on histological data.

## Introduction

The current textbook understanding of developmental and pathological processes in biological systems is mainly based on biochemical signaling. However, in recent years it has become evident that mechanical signals also play an important role in these biological processes (reviewed in (1–6)). Even in the central nervous system (CNS), which is mechanically protected from external stresses by the surrounding meninges and bones (brain and spinal cord) or fibrous tissue (retina), cells adapt their morphology, proliferation, migration, and differentiation to the stiffness of their environment (7–12). Accordingly, after introducing implants into the CNS, whose stiffness is orders of magnitude higher than that of the tissue, cells respond to the mechanical signals generated by the implant with an inflammatory reaction, culminating in a foreign body reaction (12). Mechanical signaling was also suggested to be involved in other pathological processes, such as the failure of neurons to regenerate after spinal cord injuries, or the lack of remyelination in neurodegenerative disorders, such as multiple sclerosis (5,9).

As most if not all CNS cells respond to such mechanical signals, quantitative data on the mechanical properties of CNS tissue are required for a deeper understanding of the characteristics of these mechanical signals. Rheological measurements of CNS tissue using tensile, shear, or compression methods (reviewed in (13,14)) as well as magnetic resonance elastography (reviewed in (15,16)) revealed that brain tissue is very soft, and its mechanical properties are age-dependent and heterogeneous (17–23). However, knowledge about the mechanical properties of CNS tissue at a length scale that is relevant to individual cells (approximately tens of micrometers) is currently sparse. In addition, although some CNS tissues were shown to be mechanically anisotropic, inhomogeneous, or to stiffen with strain, these studies were mostly done using different samples and methods, impeding comparability.

To overcome the constraints of bulk approaches on spatial resolution (at the millimeter scale and above), stiffness distributions of brain and retinal tissues were determined by atomic force microscopy (AFM) (24–27). However, currently there is only little information about the mechanical properties of the spinal cord. The available data have been acquired in rather macroscopic bulk measurements and are contradictory (28–36). Whereas Ichihara et al. (32) reported spinal cord gray matter to be stiffer than white matter, Ozawa et al. (35) found no difference between them.

**Figure 1:**
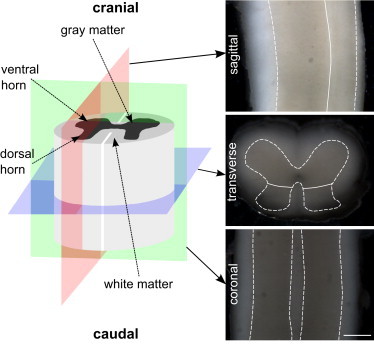
Anatomy of the spinal cord. The three anatomical planes in a schematic drawing of the spinal cord. Sagittal plane (*red*), transverse plane (*blue*), and coronal plane (*green*) are shown with their corresponding representative images of mouse spinal cord slices. White dashed and white solid lines represent the border between gray and white matter and between the ventral and dorsal horn of the gray matter, respectively. Scale bar: 500 *μ*m. To see this figure in color, go online.

Spinal cord white matter mainly consists of glial cells and long, myelinated, highly orientated axons extending along the craniocaudal (i.e., head-to-tail) axis, connecting the brain to the rest of the body. Gray matter, which is surrounded by the white matter (Fig. 1), mainly consists of neuronal cell bodies and glial cells. The most prominent regions of the spinal cord gray matter are the ventral and dorsal horns (Fig. 1). How different morphological structures contribute to local CNS tissue stiffness is currently poorly understood.

To investigate the regional and direction-dependent mechanical properties of the spinal cord at a spatial resolution relevant to individual cells (50 *μ*m), we conducted AFM indentation and pulling measurements on acutely isolated mouse spinal cord tissue cut along the three major anatomical planes (sagittal, transverse, or coronal; Fig. 1). In addition, we utilized immunohistochemistry (IHC) to correlate local mechanical tissue properties with underlying morphological structures. We found that gray matter is significantly stiffer than white matter irrespective of directionality. Furthermore, gray matter was regionally heterogeneous and anisotropic (dorsal versus ventral horn), whereas white matter was transversely isotropic under compression and isotropic under tension. Most importantly, the normalized average cell nucleus area, the cellular in plane proximity (both combined in the parameter $P_{nuclei}$; see Materials and Methods) and the orientation of neuronal axons strongly correlated with the stiffness distributions obtained by indentation experiments. Based on these data, we derived a linear model that predicts local CNS tissue stiffness based on easily accessible histological parameters. Thus, our experiments may provide the foundation for the estimation of stiffness distributions in tissues based on its morphological structure.

## Materials and Methods

### Solution and sample preparation

#### Solutions

All chemicals were obtained from Sigma Aldrich (Sigma-Aldrich Company, Ltd., Gillingham, UK) unless stated differently. The slicing artificial cerebrospinal fluid (s-aCSF) was composed of 191 mM sucrose, 0.75 mM K-gluconate, 1.25 mM KH_2_PO_4_, 26 mM NaHCO_3_, 4 mM MgSO_4_, 1 mM CaCl_2_, 20 mM glucose, 2 mM kynurenic acid, 1 mM (+)-sodium L-ascorbate, 5 mM ethyl pyruvate, 3 mM myo-inositol, and 2 mM NaOH. The measuring artificial cerebrospinal fluid (m-aCSF) was composed of 121 mM NaCl, 3 mM KCl, 1.25 mM NaH_2_PO_4_, 25 mM NaHCO_3_, 1.1 mM MgCl_2_, 2.2 mM CaCl_2_, 15 mM glucose, 1 mM (+)-sodium L-ascorbate, 5 mM ethyl pyruvate, and 3 mM myo-inositol. Both solutions were prepared freshly before each experiment and saturately bubbled with 95% O_2_/5% CO_2_. The resulting pH was ∼7.3 (37).

#### Dissection

Fourteen adult (older than 3 months) female C57BL/6 wild-type mice (indentation experiments) and seven adult male C57BL/6 wild-type mice (pulling experiments) were sacrificed by a preserved method of cervical dislocation in accordance with regulations issued by the Home Office of the United Kingdom under the Animals (Scientific Procedures) Act of 1986. To ensure the viability of the tissue, we adapted a protocol used in spinal cord electrophysiology (37) and minimized the time post mortem; elastic properties of rat brains have been shown to be similar in vivo and, within a few hours, ex vivo (38). Mice were fixed on an ice-cold aluminum wrapped polystyrene pad and then eviscerated to expose the vertebral column ventrally. The whole abdomen was washed and filled with ice cold s-aCSF, which was exchanged every 2 min. The time between cervical dislocation and first contact with s-aCSF was ∼8 min. The spinal cord was exposed and visualized under a stereo microscope. The ventral and dorsal roots and part of the meninges were cut from cranial to caudal, while the spinal cord was gently lifted up at the cranial end of the exposed area. The spinal cord was transferred to an s-aCSF–filled petri dish. Again, the s-aCSF was exchanged every 2 min. Under a stereo microscope the dura mater was carefully removed. Furthermore, the remainings of the roots were cut off. An aluminum-foil cube (2 × 2 × 2 cm^3^) was half filled with 37°C of 4% low melt agarose in 0.1 M phosphate buffered saline solution. Thereafter, the lumbar enlargement of the spinal cord was placed inside the cube, and the cube was carefully filled up with 4% low melt agarose. The cube was directly placed on ice for hardening of the agarose. This procedure was carried out within less than 30 min after sacrificing.

For immunohistochemical staining, three female C57BL/6 mice were sacrificed by CO_2_ and perfused with 4% paraformaldehyde/0.1 M phosphate buffered saline solution. The tissue was embedded in paraffin and 5 *μ*m thick sections were obtained.

#### Slice preparation

The spinal cord-containing agarose block was glued on the vibratome (VT1000 S, Leica Microsystems, Ltd, Milton Keynes, UK) plate with superglue, so that transverse, coronal, or sagittal slices could be obtained (Fig. 1). The plate was placed in an s-aCSF-filled vibratome baisin surrounded by ice. The s-aCSF was bubbled with 95% O_2_/5% CO_2_ during the whole slicing procedure. One-half of a double-edged carbon steel blade (Wilkinson Sword, Ltd, High Wycombe, UK) was positioned at an angle of 20° to the horizontal plane, the vibratory amplitude was set to 1 mm with a frequency of 100 Hz. The forward speed was set to ∼40 *μ*m/s. The slice thickness was 500 *μ*m. For AFM measurements, one slice was transferred to a petri dish, which was coated with BD Cell-Tak Cell and Tissue Adhesive (Cell-Tak; BD Biosciences, Oxford, UK). The slice was then covered with m-aCSF, which was constantly renewed by bubbled m-aCSF by a custom-built flow system with a flow rate of ∼0.33 mL/min. Before the AFM measurement started, the slice was left to recover in m-aCSF for ∼15 min.

### Atomic force microscopy

As we wanted to obtain regularly spaced, high-resolution (50 *μ*m), and at the same time large elasticity maps covering (almost) complete spinal cord cross-sections, AFM indentation measurements were chosen to measure elasticity in compression. To perform compression and tensile measurements at an equal length scale, we also used an AFM-based approach to test the tissue’s response to tensile forces. All AFM measurements started within 1 to 2 h after cervical dislocation and were done within 6 to 7 h ex vivo, during which the mechanical properties of CNS tissue do not change (24,39).

#### Setup

A JPK Nanowizard Cellhesion 200 (JPK Instruments AG, Berlin, Germany), which was set up on an inverted optical microscope (Axio Observer.A1, Carl Zeiss Ltd., Cambridge, UK) was used. The spring constant of the tipless silicon cantilevers (Arrow-TL1, NanoWorld, Neuchatel, Switzerland) was determined via the thermal noise method (40) and cantilevers with a spring constant between 0.01 and 0.02 N/m were selected. Afterward monodisperse polystyrene beads (d=(37.28±0.34)μm; microParticles GmbH, Berlin, Germany) were glued to the cantilevers. For creep experiments, cantilever beads were coated with Cell-Tak and then air-dried. The slice was transferred to the AFM-setup, and an image of the section was taken with a CCD camera (The Imaging Source, Bremen, Germany) from above (Fig. 1).

#### Indentation experiments

**Figure 2:**
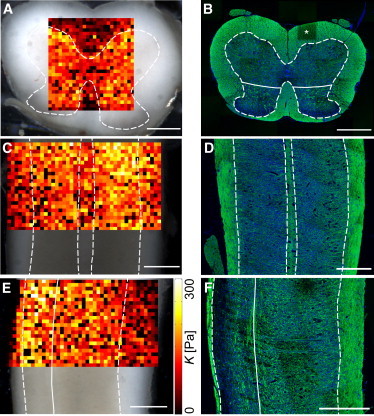
Elasticity and immunohistochemistry maps of the spinal cord. Elasticity maps and their corresponding IHC stainings (myelin: *green*; cell nuclei: *blue*) of transverse (*A* and *B*), coronal (*C* and *D*), and sagittal sections (*E* and *F*). White dashed and white solid lines represent the border between gray and white matter and between the ventral and dorsal horn, respectively. *K* is shown for full indentation; the larger *K*, the stiffer the tissue. Black squares in the elasticity map indicate missing *K* values due to unsuccessful measurements. Asterisks represent imaging artifact. Scale bars: 500 *μ*m. To see this figure in color, go online.

On the spinal cord slice, a region containing gray and white matter (Figs. 1 and 2) was selected. Force-distance curves were automatically taken every 50 *μ*m in a raster scan using a custom-written script (Fig. S1 *A* in the Supporting Material; the maximum force was 7 nN (still allowing the approximation of the probe as a paraboloid indenter), approach speed was 10 *μ*m/s (as fast as possible to enable maximum spatial resolution of the maps, but slow enough to avoid cantilever deflection because of drag), and data rate was 1000 Hz). Images of the upper-right and lower-left corners of the selected region were taken to identify the measured region (24).

#### Creep experiments

On each spinal cord section, around 20 measurements were taken on gray and white matter. The order of gray and white matter measurements was randomized to avoid selection bias. After the cantilever approached the surface, a force of 7 nN was maintained for 90 s to allow the probe to adhere to the sample. Subsequently, the cantilever was retracted at 10 *μ*m/s until a pulling force of -1 nN was reached (which is comparable to the tension along a neurite (41)), which was maintained for 30 s (Fig. S2). Although we cannot exclude an effect of the compression before the creep test on mechanical tissue properties, this effect would likely be minor, as varying the contact time before applying tension did not change the results (data not shown). Each cantilever was used for four to ten measurements.

### Immunohistochemistry

#### Staining protocol

For immunohistochemistry antigen retrieval was performed using 0.1 M citrate buffer (pH 6.0). Sections were then washed with 0.1 M tris-buffered saline (TBS; pH 7.6), and blocked for 2 h with blocking reagent (MOM kit, Vector Laboratories, Burlingame, CA). Sections were again washed with TBS, incubated for 5 min with diluent solution (MOM kit), and subsequently incubated for 30 min with the primary antibody. The primary antibodies used were SMI-99, a monoclonal antibody against myelin basic protein (MBP) (Covance, Princeton, NJ) and SMI-312, a pan-axonal neurofilament marker (Covance). All primary antibodies were diluted 1:1000 in diluent solution (MOM kit). Consecutively, sections were washed with TBS and incubated for 10 min with the secondary antibody biotin-conjugated mouse anti-mouse IgG (1:250 in diluent solution; MOM kit). The unbound secondary antibody was washed off with TBS, and sections were incubated for 45 min with NeutrAvidin 549 Dylight (1:300 in TBS, Pierce, Rockford, IL), washed again, and incubated for 10 min with 4’,6-diamidino-2-phenylindole stain solution (DAPI, Sigma-Aldrich, Schnelldorf, Germany; diluted 1:1000 in TBS). Finally, sections were washed and mounted in Aqua-PolyMount (Polysciences, Warrington, PA).

#### Imaging

All imaging was performed on a confocal microscope (TCS SP8 gSTED, Leica Microsystems, Inc.). For MBP-stained slices overview images were acquired by automatic stitching of 40× images (Leica LAF AS, Leica Microsystems, Inc.). For the neurofilament staining two to four slices for each direction (transverse, coronal, sagittal) were selected and three to four images each of the dorsal horn, ventral horn, and white matter were taken (60× magnification).

### Data analysis

All data analysis was performed with custom-written algorithms based in MATLAB (The MathWorks, Natick, MA).

#### Indentation experiments

Force-distance curves were analyzed automatically using a custom algorithm (24) based on fitting the data with the following Hertz model (42):

(1) $$F=\frac{4}{3}\frac{E}{1-\nu^{2}}r^{1/2}\delta^{3/2}=\frac{4}{3}Kr^{1/2}\delta^{3/2},$$

where the applied force is F, Young’s modulus is E, Poisson’s ratio is ν, indenter radius is r, indentation depth is δ, and the apparent reduced elastic modulus is $K=E/\left(1-\nu^{2}\right)$ (26). The curves were analyzed for different indentation depth (2, 2.5, 3, and 3.5 *μ*m) and for the full indentation depth at *F* = 7 nN (Fig. S1 *A*). Each indentation experiment was mapped onto the image of the slice (24), resulting in elasticity maps as shown in Fig. 2, *A*, *C*, and *D*. For further analysis, regions of interest (white matter, gray matter, dorsal horn, and ventral horn) were segmented by a custom-written MATLAB routine, and data within each region pooled.

#### Creep experiments

The first part of the creep experiment (Fig. S2), which is the same as the approach phase of an indentation experiment, was analyzed to obtain the contact point. We used the contact point to estimate the contact area $A_{contact}$ (which was assumed to be constant) for the actual pulling part of the experiment by $A_{contact}\approx 2\pi r\delta$, with the bead radius *r*, and the indentation depth δ at the start of the pulling part. Thereafter the Kelvin-Voigt model was fitted to the pulling part of the experiment (Fig. S2; last part) in the following:

(2) $$\varepsilon \left(t\right)=\frac{F}{A_{contact}\times E}\left(1-e^{-E/\eta \cdot t}\right),$$

where the strain is ε (assumed as 0 at the start of the creep curve), the time is *t*, and the viscosity is η (Fig. S1 *B*).

As the Poisson’s ratio of spinal cord tissue is unknown, we present the indentation and creep experiment data as *K* and *E*, respectively, which represent different parameters that were obtained at different timescales.

#### Immunohistochemistry

Overview images of MBP were processed with ImageJ software for better visualization. In each image taken from neurofilament and DAPI-stained slices, the cell number N and the *i*th cell nucleus area $A_{nucleus,i}$ were obtained by manual segmentation of all cells with a custom-written MATLAB routine. Furthermore the axon orientation Θ was set to -1 if axons were predominantly orientated perpendicularly to the optical plane (e.g., see white matter in Fig. 5 *C*), to 1 if axons were predominantly orientated parallel to the optical plane (e.g., see white matter in Fig. 5 *A*), and to 0 if orientations were mixed (e.g., see ventral horn in Fig. 5 *C*). From the cell number N and the nucleus area $A_{nucleus,i}$ of all cells, the percentage of the total image area covered by the cells’ nuclei $P_{nuclei}$ was calculated by the following:

(3) $$P_{nuclei}=\frac{\sum_{i=1}^{N}A_{nucleus,i}}{A_{image}},$$

where the total image area is $A_{image}$. In other words, $P_{nuclei}$ combines the normalized average cell nucleus area and the cellular in plane proximity.

#### Statistical analysis

Data were tested for normal distribution by the Lilliefors test; significance was tested either with Student’s *t*-test (in case of normal distribution) or the Wilcoxon rank sum test (in case of non-normal distribution). Asterisks over black line in figures indicate different significance levels: ^∗^*p* < 0.05; ^∗∗^*p* < 0.01; ^∗∗∗^*p* < 0.001.

## Results

### Gray matter is stiffer than white matter

We first assessed the mechanical properties of the spinal cord using AFM indentation experiments on spinal cord slices cut along the three major anatomical planes (transverse, coronal, or sagittal plane). Fitting the Hertz model to force-distance curves yielded an apparent reduced elastic modulus $K=E/\left(1-\nu^{2}\right)$ for each location (Fig. 1). *K* is related to two material properties; the Young’s modulus *E*, which is a measure of elastic stiffness (material stiffness increases with *E*), and the Poisson’s ratio ν, which relates the change in length of a material to its change in cross-section.

**Figure 3:**
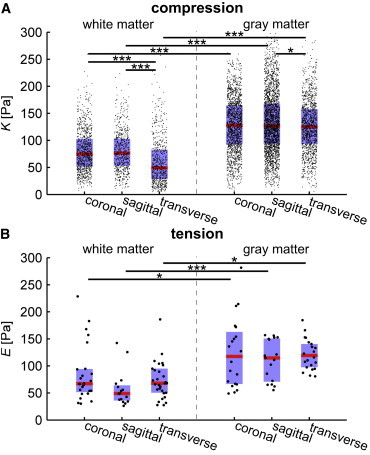
Mechanical heterogeneity and anisotropy of the spinal cord. Combined box and jittered scatter plots of *K* from indentation (*A*) and *E* from creep experiments (*B*) of white and gray matter for the three anatomical directions. Red line, blue box, and black dots represent the median, Q_1_-Q_3_ percentile, and single data points, respectively. (*A*) Apparent reduced elastic modulus *K* for full indentation of five coronal (*n*_*g*_ = 1622, *n*_*w*_ = 902), five sagittal (*n*_*g*_ = 2514, *n*_*w*_ = 623), and five transverse sections (*n*_*g*_ = 1261, *n*_*w*_ = 709). (*B*) The elastic modulus *E* of white and gray matter for coronal (two sections, *n*_*g*_ = 20, *n*_*w*_ = 24), sagittal (two sections, *n*_*g*_ = 17, *n*_*w*_ = 15) and transverse sections (three sections, *n*_*g*_ = 22, *n*_*w*_ = 30). ^∗^*p* < 0.05; ^∗∗^*p* < 0.01; ^∗∗∗^*p* < 0.001. To see this figure in color, go online.

We performed these indentation experiments as a raster scan, yielding two-dimensional elasticity maps. Representative maps are shown in Fig. 2, *A*, *C*, and *E*, for transverse, coronal, and sagittal sections, respectively. For better identification of white and gray matter, IHC overview images for MBP (showing myelinated axons) and DAPI (showing cell nuclei) are displayed in Fig. 2, *B*, *D*, and *F*. In all three anatomical planes, gray matter was significantly stiffer than white matter (about twice as stiff) at all tested indentation depths as well as for full indentation, i.e., at maximum force (*N* = 5, *n* > 600, *p* < 10^−76^) (Fig. 3 *A*). Differences in K between different animals were comparably small (Fig. S3 *A*), average *K* of gray and white matter for full indentation was ∼130 Pa and ∼70 Pa, respectively.

### Spinal cord tissue shows strain-stiffening

Comparison of stiffness distributions at different indentation depths revealed that both gray and white matter in all directions exhibited strain-stiffening (exemplary for the coronal plane, see Fig. S5). However, the stiffening followed different rules. Although the slope of strain-stiffening was higher at low indentations for gray matter compared with white matter, the strain-stiffening got more pronounced with larger indentations for white matter; for gray matter strain-stiffening got less pronounced with larger indentations (Fig. S5, *E* and *F*).

### As a whole, gray and white matter behave like an isotropic and transversely isotropic material, respectively

Cutting spinal cord tissue along all three anatomical planes enabled us to investigate if its mechanical properties are direction-dependent (Figs. 2 and 3 *A*). When data points were pooled across whole elasticity maps, K of gray matter was very similar for all slicing directions (*p* > 0.01); median K at full indentation was 128, 127, and 125 Pa for coronal (*n* = 1622), sagittal (*n* = 2514), and transverse (*n* = 1261) planes, respectively. However, the distribution of *K* was significantly broader for the sagittal plane compared with the other two planes (*p* < 10^−6^; two sample F-test on full indentation; disregarding outliers *K* > 350 Pa); regional differences were even visible in sagittal stiffness maps (see the dorsal and ventral horn in Fig. 2 *E*). Our data thus suggest that, at comparably low strains, gray matter, as a whole, behaved predominantly like an isotropic material.

However, *K* of transversely cut white matter was significantly smaller than that of the coronal and sagittal sections (*p* < 10^−16^; Fig. 3 *A*). There was no difference between K of coronally and sagittally cut white matter (*p* > 0.5), which are both longitudinal cuts along the major axon tracts. Median *K* values of white matter at full indentation were 75, 77, and 48 Pa for coronal (*n* = 902), sagittal (*n* = 623), and transverse (*n* = 667) planes, respectively (Table 1). We did not observe any significant subregional differences. Thus, the spinal cord’s white matter behaved like a transversely isotropic material.

### Under tension, gray and white matter behave like isotropic materials

To test if the mechanical behavior of spinal cord gray and white matter is different under tension compared with compression, we performed AFM creep experiments. The AFM probe was attached to the tissue slice, and the cantilever then pulled away from the tissue to apply a constant force, while the deformation of the tissue was monitored and analyzed. We found significant differences between the elastic moduli *E* of gray (*n* = 17 to 22) and white matter (*n* = 15 to 30) in all three anatomical planes (*p* < 0.05; Fig. 3 *B*); as in the indentation measurements gray matter was about twice as stiff as white matter. Furthermore, elastic moduli of gray and white matter were independent of the anatomical plane, indicating that under small tensions both gray and white matter behave predominantly isotropic. The median elastic modulus *E* of gray and white matter was 118, 115, and 119 Pa and 67, 50, and 68 Pa for the coronal (*n*_*g*_ = 20, *n*_*w*_ = 24), sagittal (*n*_*g*_ = 17, *n*_*w*_ = 15), and transverse (*n*_*g*_ = 22, *n*_*w*_ = 30) planes, respectively (Fig. 3 *B*). The median viscosity *η* of gray and white matter was 932, 827, and 938 Pa ⋅ s and 538, 441, and 497 Pa ⋅ s for the coronal, sagittal, and transverse planes, respectively (Fig. S4).

### Gray matter is stiffer in the dorsal than in the ventral horn in sagittal, but not in transverse, sections

**Figure 4:**
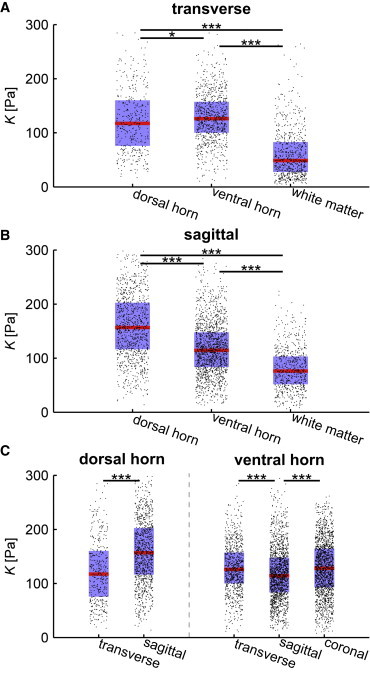
Mechanical heterogeneity of the spinal cord gray matter. Combined box and jittered scatter plots of *K* for full indentation of the dorsal horn (*n* = 402), ventral horn (*n* = 836), and white matter (*n* = 709) in the transverse plane (*A*), of the dorsal horn (*n* = 1021), ventral horn (*n* = 1535), and white matter (*n* = 623) in the sagittal plane (*B*) and the comparison of the dorsal and ventral horn dependent on anatomical plane (*C*). Red line, blue box, and black dots represent the median, Q_1_-Q_3_ percentile, and single data points, respectively. ^∗^*p* < 0.05; ^∗∗^*p* < 0.01; ^∗∗∗^*p* < 0.001. To see this figure in color, go online.

Gray matter in the spinal cord can be divided into different subregions. Two of the most prominent regions are the dorsal and ventral horns (Fig. 1), which contain the motor and sensory neurons, respectively. In coronal sections, the dorsal horn was not accessible because of the slicing procedure. In transverse sections, we observed a slightly lower *K* of gray matter of the dorsal (*n* = 402) compared with the ventral horns (*n* = 836; *p* = 0.017) (Fig. 4 *A* and Table 1). In sagittal sections, however, the dorsal horn (*n* = 1021) was significantly stiffer than the ventral horn (*n* = 653; *p* < 10^−33^) (Fig. 4 *B*), explaining the significantly broader distribution of K in the sagittal compared with the coronal and transversal sections mentioned above. In agreement with our previous results (Fig. 3 *A*), in both directions, gray matter (dorsal or ventral horn) was significantly stiffer than white matter (*n* = 1535; *p* < 10^−60^). As above, this mechanical difference was very reproducible and found in all tested animals (Fig. S3 *B*). The median apparent reduced elastic modulus of the dorsal horn, ventral horn, and white matter for transversal and sagittal sections at full indentation was 117, 126, and 48 Pa and 157, 115, and 77 Pa, respectively (Table 1). Thus, regionally the apparent reduced elastic modulus of gray matter is anisotropic and heterogeneous, which is in contrast to the mechanical behavior of gray matter analyzed as a whole (Fig. 3 *A*).

### The gray matter’s ventral horn behaves like a transversely isotropic material

We then analyzed our measurements in all three major anatomical planes for the ventral horn and in two anatomical planes for the dorsal horn. The latter was significantly softer in the transverse compared with the sagittal plane (*p* < 10^−21^) (Fig. 4 *C*). The ventral horn was significantly softer in the sagittal compared with both other directions (*p* < 10^−7^), whereas we did not observe a difference between the transverse and coronal plane (*p* > 0.05) (Fig. 4 *C*). Thus, the ventral horn behaved like a transversely isotropic material.

### Mechanical properties of the spinal cord are correlated with the distribution and sizes of cell nuclei as well as axonal directionality

**Figure 5:**
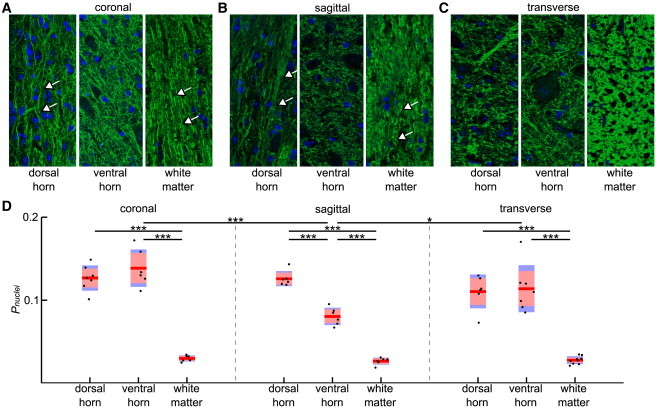
Morphology of the spinal cord. (*A–C*) IHC staining for neurofilaments and cell nuclei for coronal (*A*), sagittal (*B*), and transverse planes (*C*). (*A* and *B*) Cranial is top; caudal is bottom. White arrows indicate long axons. (*A–C*) Predominantly craniocaudally orientated neurofilaments in the dorsal horn and white matter; mixed orientation of the neurofilaments in the ventral horn. Scale bar: 50 *μ*m. (*D*) Combined box and jitter scatter plot of $P_{nuclei}$ for the dorsal horn, ventral horn, and white matter for the coronal, sagittal, and transverse planes. Red line, red box, blue box, and black dots represent the mean, SE, SD, and single data points, respectively. ^∗^*p* < 0.05; ^∗∗^*p* < 0.01; ^∗∗∗^*p* < 0.001. To see this figure in color, go online.

To explain the differences between the mechanical properties of gray matter dorsal horn, ventral horn, and white matter under compression, and in particular their direction-dependent mechanical properties, we performed IHC stainings on coronal (Fig. 5 *A*), sagittal (Fig. 5 *B*), and transverse sections (Fig. 5 *C*). We stained for phosphorylated neurofilaments and cell nuclei, to visualize axonal directionality, cell number, and approximate areas of cell nuclei. In the white matter, most axons had a craniocaudal orientation (i.e., along the spinal cord) (Fig. 5, *A*–*C*, *last subpanel*, and Table 1), strongly correlating with the observed transversely isotropic behavior of the white matter. In the ventral horn of the gray matter, axons did not have a clear directional bias (Fig. 5, *A*–*C*, *middle subpanel*, and Table 1). However, in the dorsal horn, most axons were also preferentially oriented craniocaudally (Fig. 5, *A*–*C*, *first subpanel*, and Table 1). To account for the influence of cell number and size on the mechanical properties of the spinal cord, we calculated the percentage of the tissue’s area of interest covered by cell nuclei $P_{nuclei}$. In both white matter and dorsal horn of the gray matter, $P_{nuclei}$ was not direction-dependent. In the ventral horn, however, $P_{nuclei}$ was significantly smaller in the sagittal section compared with both other directions (*p* < 0.05), likely explaining the observed transversely isotropic behavior of the ventral horn; no difference in $P_{nuclei}$ between corresponding regions in the transverse and coronal planes was observed (Fig. 5 *D*).

**Figure 6:**
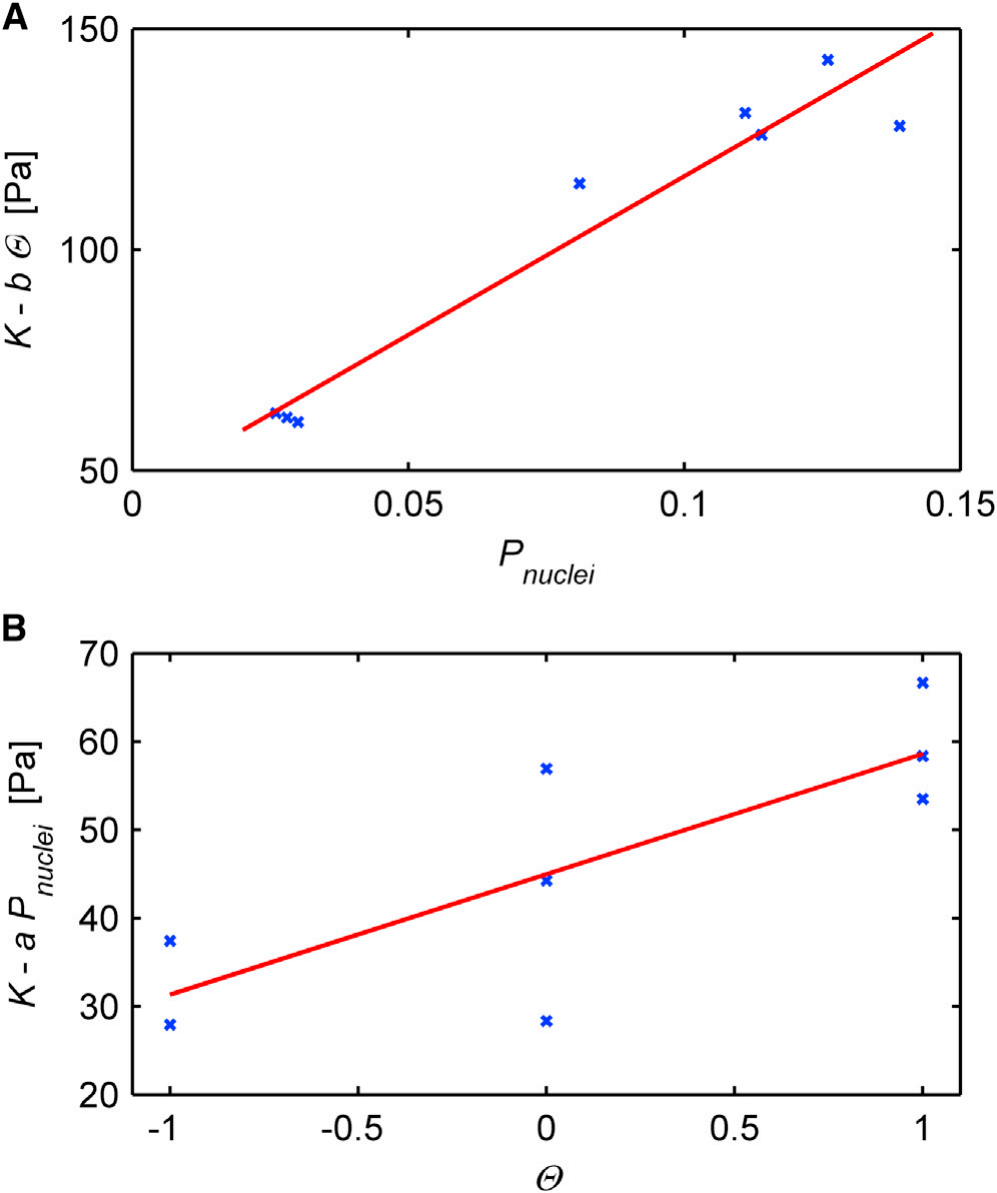
Local spinal cord tissue stiffness depends on cell distribution and axon orientation. (*A* and *B*) Semi-independent linear correlations; *K* reduced by the influence of Θ and $P_{nuclei}$ versus $P_{nuclei}$ and Θ, respectively. Blue crosses and red lines represent single data points and linear fits. The Pearson’s linear correlation coefficient is 0.97 (*A*) and 0.79 (*B*). To see this figure in color, go online.

In summary, we found a strong correlation between local tissue stiffness measured under compression and its underlying morphological structure. A higher $P_{nuclei}$ resulted in a higher *K*, whereas an axon orientation along the force direction of the indentation yielded a lower *K*, an axon orientation perpendicular to the force direction yielded in a higher *K*, and unoriented axons did not change *K* (Figs. 3, 4, 5, and 6 and Table 1).

### Prediction of CNS tissue mechanical properties using histological parameters

To test whether a prediction of the mechanical behavior of the spinal cord is possibly based on the normalized average cell nucleus area, cellular in plane proximity (both combined in the parameter $P_{nuclei}$) and axon orientation Θ, we combined these in a simple phenomenological linear model. Finding the least square fit gives an approximation of an apparent reduced elastic modulus based on the morphology of the tissue as in the following:

(4) $$K_{c}=a\times P_{nuclei}+b\times \Theta +c,$$

where *a*, *b*, and *c* are constants that depend on measurement parameters such as strain frequency and amplitude (see Fig. S5 and (5)), as well as on age, gender, and the composition of the extracellular matrix (ECM). For the median values of all our measurements at full indentation, *a* = 717 Pa, *b* = 14 Pa, and *c* = 45 Pa yielded the best fit. Semi-independent plots of $P_{nuclei}$ and Θ show reasonable good linear fits with a Pearson’s linear correlation coefficient of 0.97 and 0.79, respectively (Fig. 6). To further validate our model and to get an approximation of the accuracy, we applied our model to predict *K* for each matter and anatomical plane. For this we used the data for all other directions and matters to obtain the predicted *a*, *b*, and *c* values and therefore the predicted *K*_*c*_. All predicted *K*_*c*_ are similar to the measured median values (Table 1) with a mean ± SE deviation of (10.6 ± 2.7) Pa (relative mean ± SE: (10.6 ± 2.6) %).

Applying the full model with the obtained parameters *a* = 717 Pa, *b* = 14 Pa, and *c* = 45 Pa to the dorsal horn in coronal sections—which we did not measure by AFM—returns *K*_*c*_ = 150 Pa, which is similar to the *K* value in the sagittal plane. Therefore, we predict that the dorsal horn behaves like a transversely isotropic material, with K being smaller in the transverse compared with the sagittal and coronal plane (Table 1).

## Discussion

We systematically studied the direction-dependent mechanical properties of adult mouse spinal cord tissue in the three major anatomical planes with 50 *μ*m resolution and found that its mechanical behavior is highly complex, that it is region and direction-dependent, and that it strongly correlates with the underlying cellular structures. Although gray matter as a whole behaved isotropically under compression, gray matter subregions were rather inhomogeneous and anisotropic. For example, in sagittal sections the dorsal horn was significantly stiffer than the ventral horn. In contrast, white matter under compression behaved as a transversely isotropic material, with the elastic stiffness along the craniocaudal (i.e., longitudinal) axis being lower than perpendicular to that axis. Under small tension, however, both gray and white matter behaved like an isotropic material. In all cases, gray matter was about twice as stiff as white matter, irrespective of directionality (transverse, coronal, and sagittal plane) and force direction (compression or tension).

Stiffness differences between gray and white matter have been described before, but not without controversy. Although Christ et al., Ichihara et al., and Green et al. also found gray matter to be stiffer than white matter (24,32,43), Ozawa et al. saw no difference (35), and Kruse et al. and McCracken et al. observed gray matter to be softer than white matter (44,45). Furthermore, the elastic moduli of CNS tissue reported in the literature range between ∼50 and 20,000 Pa (5). Both apparent discrepancies may be explained by the highly complex, nonlinear, viscoelastic properties of CNS tissue; time and length scales of the mechanical tests significantly influence the results (as discussed in (5, 46)).

When assessed at time (∼s) and length scales (∼*μ*m) relevant to cell physiology, CNS tissue is mechanically heterogeneous with elastic moduli on the order of hundreds of Pascal (17,24–27). And although gray and white matter are inverted in the spinal cord with respect to the brain, gray matter is about twice as stiff as white matter in both tissues when assessed by AFM, i.e., at low strains. Although our measurements might not directly predict the response of the bulk tissue to large deformations (such as often encountered in spinal cord injuries), this study revealed mechanical heterogeneities at a cellular scale, which may be relevant for the mechanosensation of cells (5).

The stiffness of tissues in the body is often dominated by their collagen content (47). However, the ECM within the healthy CNS contains only little collagen (48), which might explain its softness and the major contribution of cells to tissue stiffness. The diffuse matrix in the adult spinal cord is homogeneously distributed and lacks link proteins (49). Thus, its contribution to CNS tissue stiffness heterogeneities is likely minor. Perineuronal nets, which are local ECM structures surrounding neurons, may vary within the spinal cord (49). Their contribution to spinal cord heterogeneity is likely included in the first term of our model, as they follow the cell distribution.

Our data indicate that differences in local mechanical tissue properties strongly correlate with the normalized average cell nucleus area and the cellular in plane proximity (i.e., $P_{nuclei}$) (Figs. 4 and 5 and Table 1); less densely packed cell bodies—as in white matter compared with gray matter—will lower the elastic stiffness of the tissue. Apparent reduced elastic moduli obtained in this study are on the order of what has been found for individual neurons and glial cells (27,50,51), suggesting that for small strains the elastic stiffness of adult CNS tissue is dominated by its constituting cells. In agreement with a dominant role of cells as major determinant of CNS tissue stiffness, the mechanical resistance of spinal cords to macroscopic stretch mainly depends on cellular structures (33,36). Longitudinally oriented spinal cord fibers were found to be significantly stronger than the surrounding matrix, providing resistance to longitudinal loading (33). Similarly, in the brain stem, axons are significantly stiffer than the ECM (52). Also oligodendrocytes and astrocytes, which contribute most cell bodies to white matter, were shown to provide stiffness and tensile strength to the spinal cord, suggesting that myelin (formed by oligodendrocytes) and cellular coupling of axons via the glial matrix affect mechanical tissue properties (36).

Neuronal cell bodies are about twice as stiff as their own processes and neighboring glial cells (50). Furthermore, cell processes of (retinal Müller) glial cells are significantly softer than their cell bodies (50). Thus, the facts that gray matter contains more (stiffer) neuronal cell bodies than white matter, and that white matter is dominated by long myelinated axon tracts, i.e., by (softer) neuronal and glial cell processes, could explain why gray matter is about twice as stiff as white matter. However, cellular stiffness alone cannot account for stiffness heterogeneities at least in brain tissue (27), suggesting that cell body density has a larger impact on local tissue stiffness than cell stiffness itself. In agreement, enhanced neurogenesis in the adult mouse, and thus an increase in cell density, is accompanied by an increase in brain stiffness (53).

Furthermore, anisotropy in CNS tissue structure because of the different orientations of the axons is likely the origin of the anisotropy in mechanical properties we found in white matter (Figs. 3 and 5). Spinal cord white matter is highly structured; it consists of parallel, densely packed, myelinated axons. Under compression it behaved like a transversely isotropic material; axon orientations along the indentation direction lowered the elastic stiffness, and axon orientations perpendicular to the indentation direction returned a higher elastic stiffness. Hence, both longitudinal planes showed similar mechanical properties, whereas the transverse plane was different. In line with our study, Feng et al. (54) found evidence for mechanical transverse isotropy of white matter in the brain.

Axons in vivo may be under tension (55). Cutting the spinal cord transversally could lead to a retraction and relaxation of the cut axons. As cell stiffness scales with prestress (or tension) (13), this loss of tension could explain why the surface of the white matter under compression, but not under tension, is softer in transverse cuts than in longitudinal ones. On the other hand, the spinal cord is frequently exposed to large strains (e.g., during bending of the back), and axons in unstretched spinal cords are undulated to accommodate stretching (56), indicating that they are relaxed even in intact tissue. Furthermore, mechanical anisotropy has been found in brain white matter using macroscopic magnetic resonance elastography tests (19,57), suggesting that spinal cord white matter anisotropy is not a mere sample preparation artifact but rather a direct consequence of the presence of highly oriented fibrillar structures (i.e., myelinated axon tracts). Because of strain stiffening of the longitudinally orientated fibers, it is likely that, at larger strains, white matter resists tension even more along the fibers than perpendicular to them (in contrast to compression).

Because gray matter is mainly comprised of cell bodies, and long axon tracts are absent, as a whole it behaves predominantly isotropically. However, subregions of the gray matter differed in the normalized average cell nucleus area, cellular in plane proximity (i.e., $P_{nuclei}$), and their predominant axon orientations. In the ventral horn, axon orientations were mixed, whereas in the dorsal horn most axons were oriented craniocaudally. In the ventral horn, $P_{nuclei}$ was lower for the sagittal than for the transverse and coronal planes, whereas there was no direction-dependent difference for the dorsal horn (Fig. 5 *D* and Table 1). Thus, $P_{nuclei}$ may explain the transverse isotropy of the ventral horn, and the axon orientation in the dorsal horn its transverse isotropy, which we predicted with our model (Fig. 4 and Table 1).

By only using the axon orientation with discrete scaling and $P_{nuclei}$, we derived a simple phenomenological model to predict approximate local direction-dependent stiffness distributions within CNS tissue under small compression from histological data (Fig. 6 and Table 1). Although our model is only a first-order approximation and does not specifically take into account exact axon orientations and densities, cell types (50), or ECM composition, our predictions were within 11% of the measured values and well within the Q_1_-Q_3_ percentile of our measurements.

Cell types, sizes, morphologies, and distributions, as well as the ECM composition may vary significantly between different regions in the CNS and across species, and they may change during development and ageing as well as in pathological conditions. As a result, the fitting parameters of our model may have to be adapted to new samples.

Histological changes are often accompanied by changes in tissue stiffness. For example, brains of patients suffering from multiple sclerosis soften by 13% to 20.5% (58,59). To more accurately estimate the mechanical properties of any given tissue based on its histology, a deeper analysis of the tissue’s structure and components is necessary. Furthermore, to yield a model with a higher predictive power and a broader usage, a continuous scale of fiber anisotropy, the actual volume, geometry, type, and three-dimensional spacing of cells, and the influence of different ECM components and their local expression levels need to be taken into account.

## Conclusions

Throughout life, cells respond to a multitude of chemical as well as mechanical signals, which determine their biological function. Mechanical signals in the CNS may change during development and pathologies (1–6). Although the mechanical differences we found between different subregions of the spinal cord are only on the order of tens of Pascal, even subtle changes in tissue stiffness may influence CNS cell behavior (9,10). Furthermore, even the slightest influence on a cell’s behavior (e.g., on its migration) will result in a biased behavior over time.

Characterizing the mechanical signals cells encounter, e.g., the mechanical properties of their surrounding tissue, is thus a crucial step in completing our picture of cell function control. However, currently available methods to measure local tissue stiffness at a length scale that is relevant to individual cells are not readily available, and measurements are complex and time-consuming.

In this study we combined a detailed characterization of cell distributions and axon orientations in the adult mouse spinal cord with the assessment of local spinal cord tissue stiffness via AFM. Our study revealed a strong correlation between the ultrastructure and mechanical properties of spinal cord tissue (Fig. 6 and Table 1). We incorporated this relationship into a phenomenological model (Eq. 4), which is a first step toward estimating local CNS tissue stiffness purely based on histological structures. Optical microscopy, which has been extensively used to study cell signaling, and which is a widespread standard technique in biology laboratories, could thus be used to approximate tissue stiffness, and to track changes in mechanical signaling during development and disorders, such as neurodegenerative diseases and traumatic brain and spinal cord injuries.

## Author Contributions

D.E.K. and K.F. designed the research; D.E.K., E.M., J.H., and S.K. performed research; D.E.K. and E.M. analyzed data; D.E.K. and K.F. wrote the article, with contributions from all co-authors.

## Figures and Tables

**Table 1:** Direction-dependent stiffness, histological parameters, and predicted stiffness of mice spinal cord

	White Matter			Ventral Horn			Dorsal Horn		
	Coronal	Sagittal	Transversal	Coronal	Sagittal	Transversal	Coronal	Sagittal	Transversal
*K* [Pa]	75	77	48	128	115	126	—	157	117
$P_{\mathrm{nuclei}}$	0.030	0.026	0.028	0.0139	0.081	0.114	0.127	0.0126	0.111
Θ	1	1	−1	0	0	0	1	1	−1
*K*_*c*_ [Pa]	84	78	58	153	101	127	150	143	106

## Supporting Material

Document S1. Five figures

Document S2. Article plus Supporting Material

## References

[1] Fletcher D.A., Mullins R.D. (2010). Cell mechanics and the cytoskeleton. Nature.

[2] Eyckmans J., Boudou T., Chen C.S. (2011). A hitchhiker’s guide to mechanobiology. Dev. Cell.

[3] Tyler W.J. (2012). The mechanobiology of brain function. Nat. Rev. Neurosci..

[4] Franze K. (2013). The mechanical control of nervous system development. Development.

[5] Franze K., Janmey P.A., Guck J. (2013). Mechanics in neuronal development and repair. Annu. Rev. Biomed. Eng..

[6] Mammoto T., Mammoto A., Ingber D.E. (2013). Mechanobiology and developmental control. Annu. Rev. Cell Dev. Biol..

[7] Flanagan L.A., Ju Y.E., Janmey P.A. (2002). Neurite branching on deformable substrates. Neuroreport.

[8] Georges P.C., Miller W.J., Janmey P.A. (2006). Matrices with compliance comparable to that of brain tissue select neuronal over glial growth in mixed cortical cultures. Biophys. J..

[9] Jagielska A., Norman A.L., Franklin R.J.M. (2012). Mechanical environment modulates biological properties of oligodendrocyte progenitor cells. Stem Cells Dev..

[10] Koch D., Rosoff W.J., Urbach J.S. (2012). Strength in the periphery: growth cone biomechanics and substrate rigidity response in peripheral and central nervous system neurons. Biophys. J..

[11] Moshayedi P., Costa L. da. F., Franze K. (2010). Mechanosensitivity of astrocytes on optimized polyacrylamide gels analyzed by quantitative morphometry. J. Phys. Condens. Matter.

[12] Moshayedi P., Ng G., Guck J. (2014). The relationship between glial cell mechanosensitivity and foreign body reactions in the central nervous system. Biomaterials.

[13] Cheng S., Clarke E.C., Bilston L.E. (2008). Rheological properties of the tissues of the central nervous system: a review. Med. Eng. Phys..

[14] Chatelin S., Constantinesco A., Willinger R. (2010). Fifty years of brain tissue mechanical testing: from in vitro to in vivo investigations. Biorheology.

[15] Mariappan Y.K., Glaser K.J., Ehman R.L. (2010). Magnetic resonance elastography: a review. Clin. Anat..

[16] Di Ieva A., Grizzi F., Rodriguez y Baena R. (2010). Magnetic resonance elastography: a general overview of its current and future applications in brain imaging. Neurosurg. Rev..

[17] Elkin B.S., Ilankovan A.I., Morrison B. (2011). A detailed viscoelastic characterization of the P17 and adult rat brain. J. Neurotrauma.

[18] Gefen A., Margulies S.S. (2004). Are in vivo and in situ brain tissues mechanically similar?. J. Biomech..

[19] Johnson C.L., McGarry M.D.J., Georgiadis J.G. (2013). Local mechanical properties of white matter structures in the human brain. Neuroimage.

[20] Pervin F., Chen W.W. (2009). Dynamic mechanical response of bovine gray matter and white matter brain tissues under compression. J. Biomech..

[21] Prange M.T., Margulies S.S. (2002). Regional, directional, and age-dependent properties of the brain undergoing large deformation. J. Biomech. Eng..

[22] Prevost T.P., Jin G., Socrate S. (2011). Dynamic mechanical response of brain tissue in indentation in vivo, in situ and in vitro. Acta Biomater..

[23] Sack I., Streitberger K.-J., Braun J. (2011). The influence of physiological aging and atrophy on brain viscoelastic properties in humans. PLoS ONE.

[24] Christ A.F., Franze K., Guck J. (2010). Mechanical difference between white and gray matter in the rat cerebellum measured by scanning force microscopy. J. Biomech..

[25] Elkin B.S., Azeloglu E.U., Morrison B. (2007). Mechanical heterogeneity of the rat hippocampus measured by atomic force microscope indentation. J. Neurotrauma.

[26] Franze K., Francke M., Guck J. (2011). Spatial mapping of the mechanical properties of the living retina using scanning force microscopy. Soft Matter.

[27] Iwashita M., Kataoka N., Kosodo Y. (2014). Systematic profiling of spatiotemporal tissue and cellular stiffness in the developing brain. Development.

[28] Bilston L.E., Thibault L.E. (1996). The mechanical properties of the human cervical spinal cord in vitro. Ann. Biomed. Eng..

[29] Fiford R.J., Bilston L.E. (2005). The mechanical properties of rat spinal cord in vitro. J. Biomech..

[30] Hung T.-K., Chang G.-L., Albin M.S. (1981). Stress-strain relationship and neurological sequelae of uniaxial elongation of the spinal cord of cats. Surg. Neurol..

[31] Hung T.-K., Chang G.-L., Bunegin L. (1981). Stress-strain relationship of the spinal cord of anesthetized cats. J. Biomech..

[32] Ichihara K., Taguchi T., Kawai S. (2001). Gray matter of the bovine cervical spinal cord is mechanically more rigid and fragile than the white matter. J. Neurotrauma.

[33] Luna C., Detrick L., Aranda-Espinoza H. (2013). Mechanical properties of the lamprey spinal cord: uniaxial loading and physiological strain. J. Biomech..

[34] Oakland R.J., Hall R.M., Barton D.C. (2006). The biomechanical response of spinal cord tissue to uniaxial loading. Proc. Inst. Mech. Eng. Part H J. Eng. Med..

[35] Ozawa H., Matsumoto T., Kokubun S. (2001). Comparison of spinal cord gray matter and white matter softness: measurement by pipette aspiration method. J. Neurosurg..

[36] Shreiber D.I., Hao H., Elias R.A.I. (2009). Probing the influence of myelin and glia on the tensile properties of the spinal cord. Biomech. Model. Mechanobiol..

[37] Mitra P., Brownstone R.M. (2012). An in vitro spinal cord slice preparation for recording from lumbar motoneurons of the adult mouse. J. Neurophysiol..

[38] Shulyakov A.V., Fernando F., Del Bigio M.R. (2009). Simultaneous determination of mechanical properties and physiologic parameters in living rat brain. Biomech. Model. Mechanobiol..

[39] Garo A., Hrapko M., Peters G.W.M. (2007). Towards a reliable characterisation of the mechanical behaviour of brain tissue: the effects of post-mortem time and sample preparation. Biorheology.

[40] Hutter J.L., Bechhoefer J. (1993). Calibration of atomic-force microscope tips. Rev. Sci. Instrum..

[41] Betz T., Koch D., Käs J.A. (2011). Growth cones as soft and weak force generators. Proc. Natl. Acad. Sci. USA.

[42] Hertz H. (1881). Über die Berührung fester elastischer Körper. J. für die reine und Angew. Math..

[43] Green M.A., Bilston L.E., Sinkus R. (2008). *In vivo* brain viscoelastic properties measured by magnetic resonance elastography. NMR Biomed..

[44] Kruse S.A., Rose G.H., Ehman R.L. (2008). Magnetic resonance elastography of the brain. Neuroimage.

[45] McCracken P.J., Manduca A., Ehman R.L. (2005). Mechanical transient-based magnetic resonance elastography. Magn. Reson. Med..

[46] McKee C.T., Last J.A., Murphy C.J. (2011). Indentation versus tensile measurements of Young’s modulus for soft biological tissues. Tissue Eng. Part B Rev..

[47] Swift J., Ivanovska I.L., Discher D.E. (2013). Nuclear lamin-A scales with tissue stiffness and enhances matrix-directed differentiation. Science.

[48] Novak U., Kaye A.H. (2000). Extracellular matrix and the brain: components and function. J. Clin. Neurosci..

[49] Galtrey C.M., Kwok J.C.F., Fawcett J.W. (2008). Distribution and synthesis of extracellular matrix proteoglycans, hyaluronan, link proteins and tenascin-R in the rat spinal cord. Eur. J. Neurosci..

[50] Lu Y.-B., Franze K., Reichenbach A. (2006). Viscoelastic properties of individual glial cells and neurons in the CNS. Proc. Natl. Acad. Sci. USA.

[51] Spedden E., White J.D., Staii C. (2012). Elasticity maps of living neurons measured by combined fluorescence and atomic force microscopy. Biophys. J..

[52] Javid S., Rezaei A., Karami G. (2014). A micromechanical procedure for viscoelastic characterization of the axons and ECM of the brainstem. J. Mech. Behav. Biomed. Mater..

[53] Klein C., Hain E.G., Sack I. (2014). Enhanced adult neurogenesis increases brain stiffness: in vivo magnetic resonance elastography in a mouse model of dopamine depletion. PLoS ONE.

[54] Feng Y., Okamoto R.J., Bayly P.V. (2013). Measurements of mechanical anisotropy in brain tissue and implications for transversely isotropic material models of white matter. J. Mech. Behav. Biomed. Mater..

[55] Siechen S., Yang S., Saif T. (2009). Mechanical tension contributes to clustering of neurotransmitter vesicles at presynaptic terminals. Proc. Natl. Acad. Sci. USA.

[56] Hao H., Shreiber D.I. (2007). Axon kinematics change during growth and development. J. Biomech. Eng..

[57] Romano A., Scheel M., Sack I. (2012). In vivo waveguide elastography of white matter tracts in the human brain. Magn. Reson. Med..

[58] Wuerfel J., Paul F., Sack I. (2010). MR-elastography reveals degradation of tissue integrity in multiple sclerosis. Neuroimage.

[59] Streitberger K.-J., Sack I., Wuerfel J. (2012). Brain viscoelasticity alteration in chronic-progressive multiple sclerosis. PLoS ONE.

