# Supplementary material for: CNS Cell Distribution and Axon Orientation Determine Local Spinal Cord Mechanical Properties
Source: Biophys J. 2015 May 5;108(9):2137–47. doi: 10.1016/j.bpj.2015.03.039 (PMC4423070; doi:10.1016/j.bpj.2015.03.039)
Supplement: Document S1. Five figures [file mmc1.pdf]

Biophysical Journal

Supporting Material

CNS Cell Distribution and Axon Orientation Determine Local Spinal Cord Mechanical Properties

David E. Koser,[1,2] Emad Moeendarbary,[1] Janina Hanne,[1] Stefanie Kuerten,[2,3] and Kristian Franze[1,]*

[1]Department of Physiology, Development and Neuroscience, University of Cambridge, Cambridge, United Kingdom; [2]Department of Anatomy I, University of Cologne, Cologne, Germany; and [3]Department of Anatomy and Cell Biology, University of Wuerzburg, Wuerzburg, Germany

SI Figures:

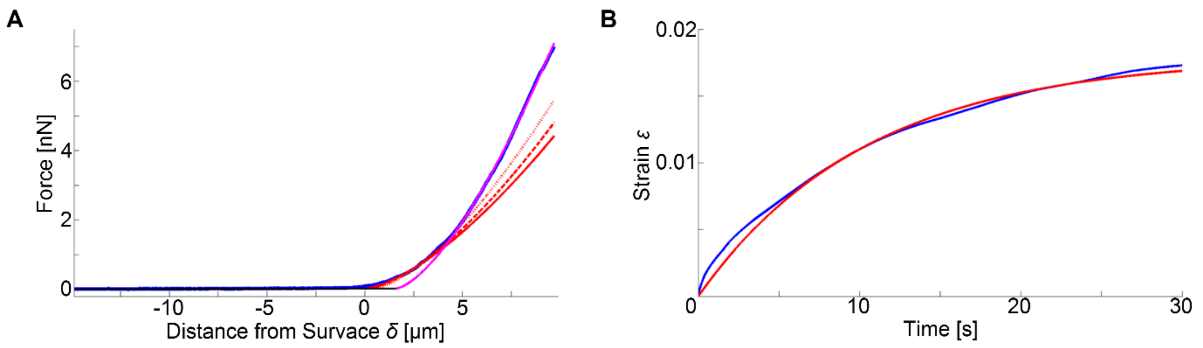

FIGURE S1 Raw indentation and creep data with fits. (*A*) Representative force-distance curve of an indentation experiment in *blue*. *Black line* shows baseline fit for each indentation. The 2 µm, 2.5 µm, 3 µm and full indentation Hertz model fits (Eq. 1) in *solid red*, *dashed red*, *doted red* and *magenta*, respectively. (*B*) Representative strain-time curve of a creep experiment in *blue*. The Kelvin-Voigt model fit (Eq. 2) in *red*.

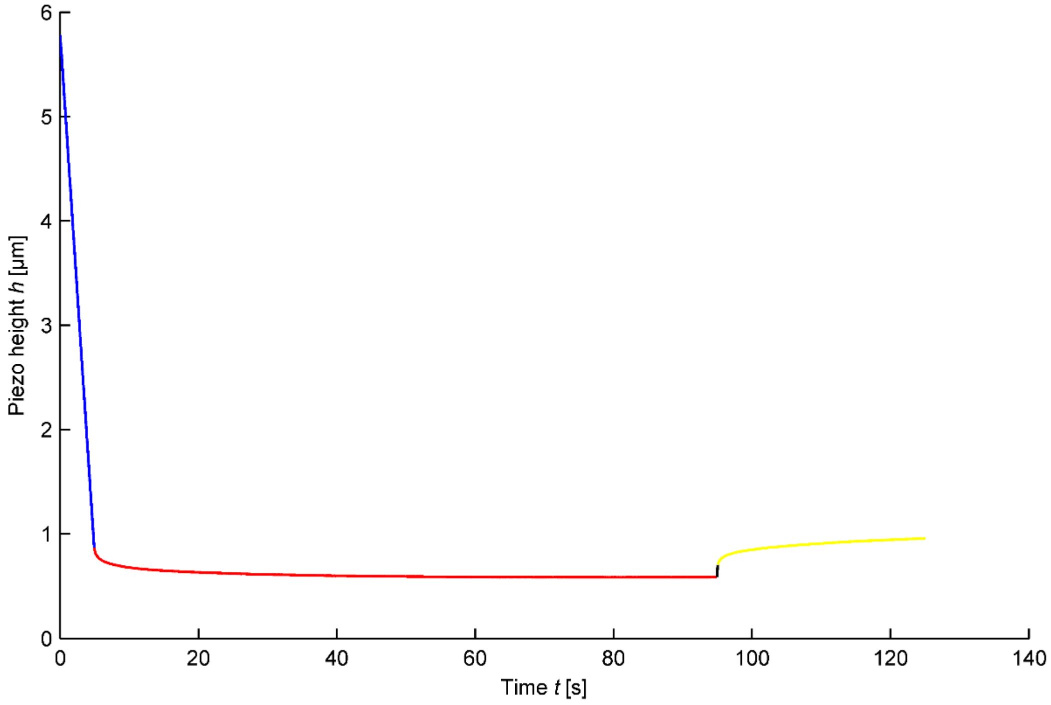

FIGURE S2 Representative height-time curve of a creep experiment. The four parts of the experiment in *blue*, *red*, *black* and *yellow*, respectively. First part: normal approach till 7 nN. Second part: 90 s constant 7 nN push-force. Third part: fast retraction till -1 nN. Fourth part: 30 s constant -1 nN pull-force (actual creep experiment; see S1 B).

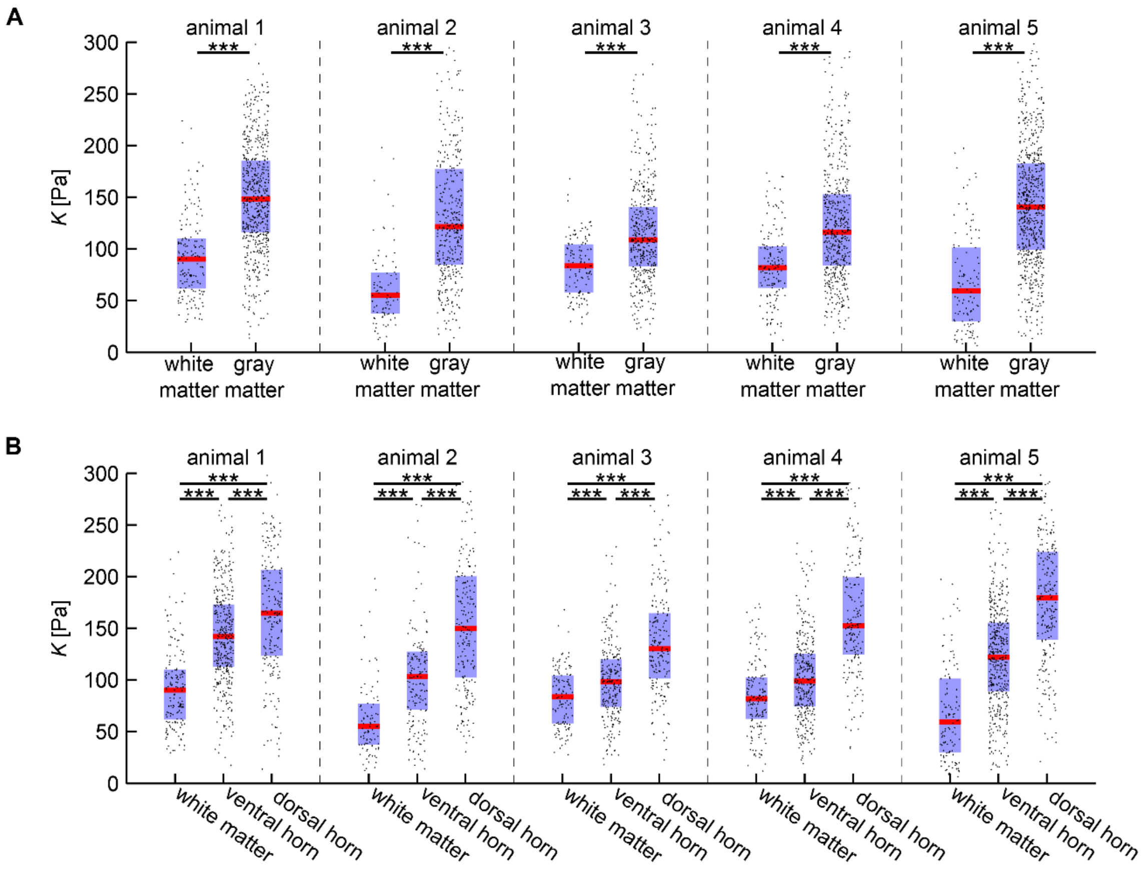

FIGURE S3 Interindividual differences of *K*. Combined box and jittered scatter plots of *K* for the sagittal sections of all 5 animals displaying the difference between white and gray matter (*A*) and dorsal horn, ventral horn and white matter (*B*). *Red line*, *blue box* and *black dots* represent the median, Q_1-Q_3 percentile, and single data points, respectively. * ($p < 0.05$); ** ($p < 0.01$); *** ($p < 0.001$).

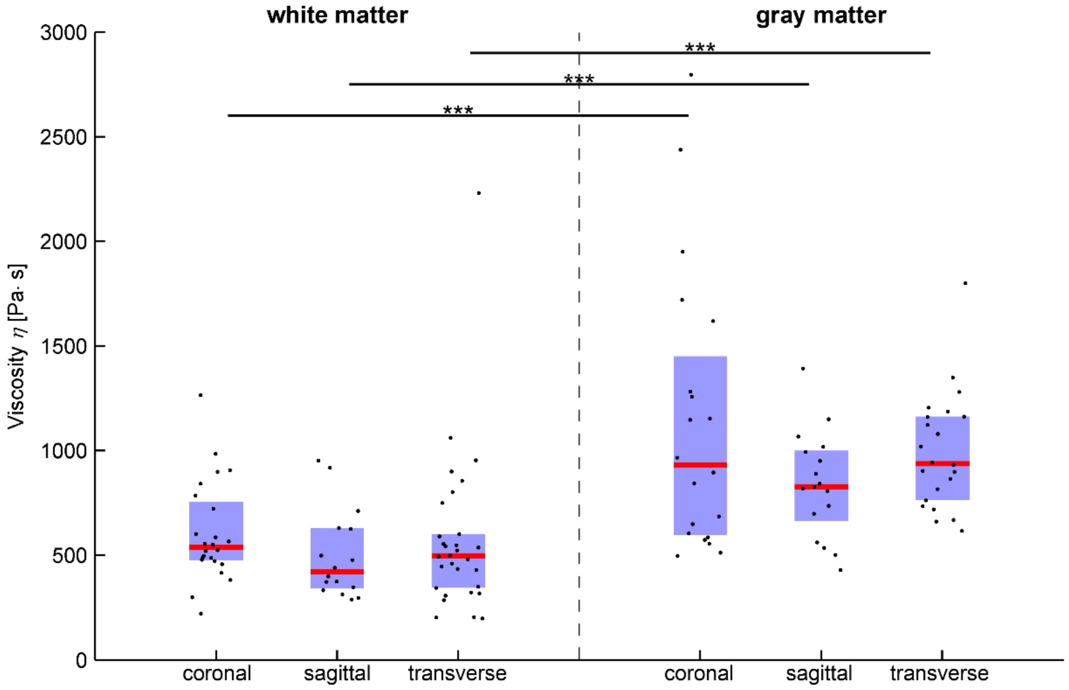

FIGURE S4 Viscosity from creep experiments. Combined box and jittered scatter plots of the viscosity η of white and gray matter for coronal (2 sections, $n_g = 20$, $n_w = 24$), sagittal (2 sections, $n_g = 17$, $n_w = 15$) and transverse sections (3 sections, $n_g = 22$, $n_w = 30$). *Red line*, *blue box* and *black dots* represent the median, Q_1-Q_3 percentile, and single data points, respectively. * ($p < 0.05$); ** ($p < 0.01$); *** ($p < 0.001$).

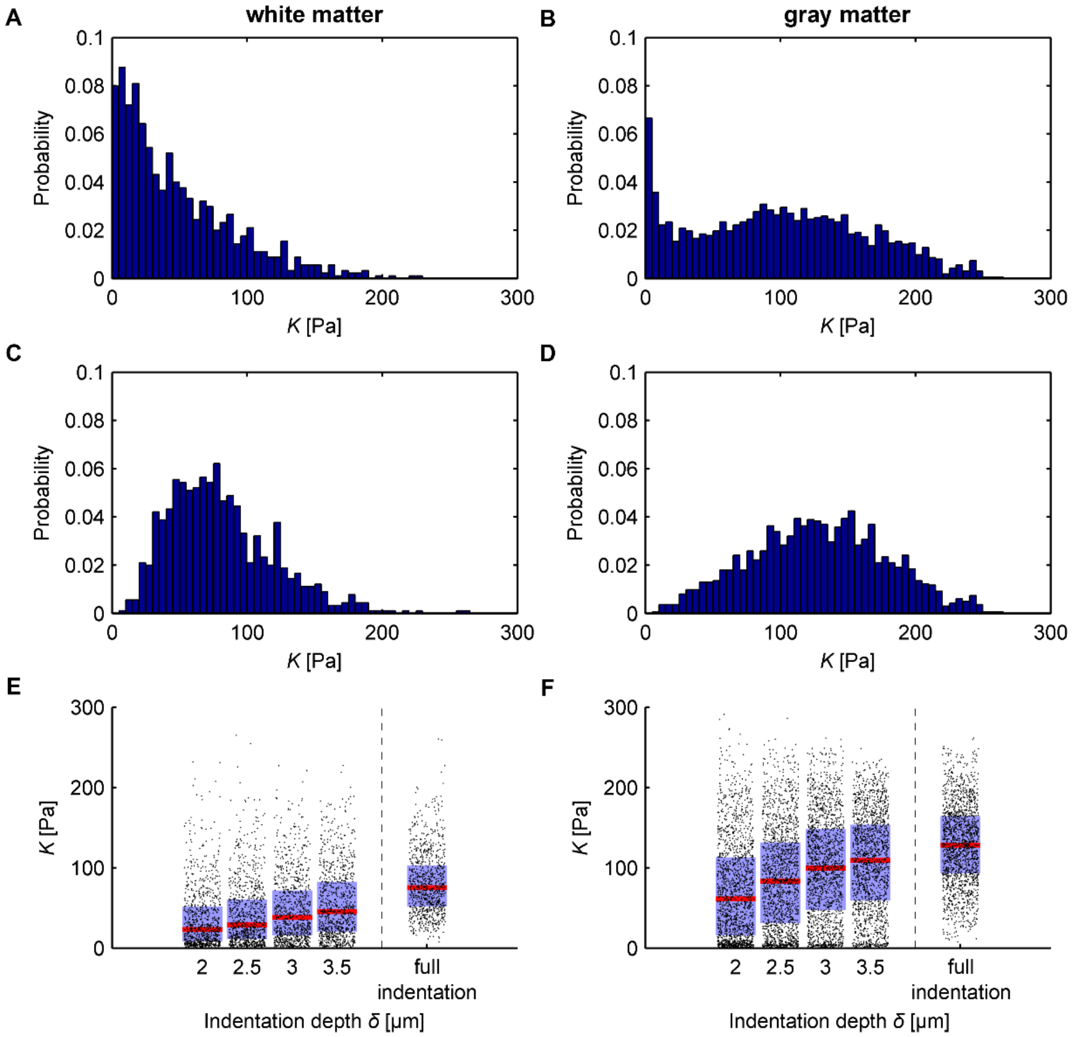

FIGURE S5 Strain stiffening of spinal cord white and gray matter. (*A*-*D*) Histograms of *K* in the coronal plane for white (*A*, *C*) and gray matter (*B*, *D*) at 3 µm (*A*, *B*) and full indentation (*C*, *D*). (*E*, *F*) Combined box and jittered scatter plots of *K* for white and gray matter for 2 µm, 2.5 µm, 3 µm, 3.5 µm and full indentation depth δ in the coronal plane. *Red line*, *blue box* and *black dots* represent the median, Q_1-Q_3 percentile, and single data points, respectively. At low indentations a shift of the median *K* towards higher values with growing indentation depth.
